# From lifetime to evolution: timescales of human gut microbiota adaptation

**DOI:** 10.3389/fmicb.2014.00587

**Published:** 2014-11-04

**Authors:** Sara Quercia, Marco Candela, Cristina Giuliani, Silvia Turroni, Donata Luiselli, Simone Rampelli, Patrizia Brigidi, Claudio Franceschi, Maria Giulia Bacalini, Paolo Garagnani, Chiara Pirazzini

**Affiliations:** ^1^Department of Pharmacy and Biotechnology, University of BolognaBologna, Italy; ^2^BiGEA, Department of Biological, Geological and Environmental Sciences, Laboratory of Molecular Anthropology & Centre for Genome Biology, University of BolognaBologna, Italy; ^3^DIMES, Department of Experimental, Diagnostic and Specialty Medicine, University of BolognaBologna, Italy; ^4^CIG, Interdepartmental Centre “L. Galvani” CIG, University of BolognaBologna, Italy; ^5^IRCSS, Institute of Neurological Sciences of BolognaBologna, Italy; ^6^IGM-CNR, Institute of Molecular Genetics, Unit of Bologna IORBologna, Italy; ^7^CNR, Institute of Organic Synthesis and Photoreactivity (ISOF)Bologna, Italy; ^8^CRBA, Center for Applied Biomedical Research, St. Orsola-Malpighi University HospitalBologna, Italy

**Keywords:** gut microbiota, aging, environmental stimuli, co-evolution, biological adaptation

## Abstract

Human beings harbor gut microbial communities that are essential to preserve human health. Molded by the human genome, the gut microbiota (GM) is an adaptive component of the human superorganisms that allows host adaptation at different timescales, optimizing host physiology from daily life to lifespan scales and human evolutionary history. The GM continuously changes from birth up to the most extreme limits of human life, reconfiguring its metagenomic layout in response to daily variations in diet or specific host physiological and immunological needs at different ages. On the other hand, the microbiota plasticity was strategic to face changes in lifestyle and dietary habits along the course of the recent evolutionary history, that has driven the passage from Paleolithic hunter-gathering societies to Neolithic agricultural farmers to modern Westernized societies.

## INTESTINAL MICROBIOTA, STRUCTURE, AND ROLE IN HUMAN PHYSIOLOGY

Human beings co-evolved as superorganisms as the result of the mutualistic relationship with the enormous microbial community that resides in human gastrointestinal tract (GIT); this ecosystem is better known as gut microbiota (GM; Turnbaugh et al., 2007). The GM reaches the highest cell concentration in the colon, with a density of 10^12^ CFU/g of intestinal content and represents the most densely populated and biodiverse ecosystem on earth (O’Hara and Shanahan, 2006; Ley et al., 2008a). The GM presents a very particular phylogenetic structure, resulting in a sparsely branched tree, with a high degree of radiation at the ends. Indeed, out of the 100 different bacterial phyla detected on our planet, only seven are found in our gut – Firmicutes, Bacteroidetes, Actinobacteria, Proteobacteria, Verrucomicrobia, Tenericutes, and Fusobacteria – of which Firmicutes and Bacteroidetes represent together up to 90% of the ecosystem (Costello et al., 2009). Conversely, the GM shows an impressive biodiversity at lower phylogenetic levels with up to 1000 different species being detected by next generation sequencing-based approaches (Qin et al., 2010). Interestingly, the species-level GM composition varies dramatically among people, and each subject owns a very unique subset of microorganisms, that consists of hundreds of the more than 1000 species detected in the GM of the entire human population. The total genome of these microorganisms, often referred to as the intestinal microbiome, has been estimated to contain 150 times more genes than the human one, providing the host with essential functional traits that human beings have not evolved on their own (Qin et al., 2010). For instance, the carbohydrate-active enzymes encoded in the microbial glycobiome allow the host to extract energy from otherwise indigestible polysaccharides (Gill et al., 2006), complementing the poor human glycobiome diversity. Indeed, the functional assignment of the gut microbiome revealed an extraordinary diversity of gene clusters involved in carbohydrate metabolism (Koropatkin et al., 2012). Moreover, the gut microbiome is enriched in genes involved in the production of vitamins, cofactors, and secondary metabolites, further supporting its important role in host nutrition (Bäckhed et al., 2004; Clemente et al., 2012). The GM is also an active component of the human immune system (Round and Mazmanian, 2009; Maynard et al., 2012). The cross-talk with intestinal microbes has been shown to be essential for the maturation of a correct immune function in early life and to preserve a well-balanced immune homeostasis later in life (Kamada et al., 2013). Finally, a new and only sparsely explored role of the GM in human physiology is its potential to modulate the function of the gut-brain axis. Indeed, accumulating data from studies carried out in mouse models suggest how the GM and its metabolites could affect the behavior and pain, in addition to depression, anxiety and other disorders belonging to the central nervous system (Cryan and Dinan, 2012). Gut commensals are capable of activating neural pathways and modulating signaling to the central nervous system through their metabolite production (Foster and McVey Neufeld, 2013). In particular, studies carried out on mice demonstrated a strategic role for commensal bacteria in programming the hypothalamic–pituitary–adrenal (HPA) stress responsiveness at early developmental stages, when brain plasticity is still preserved (Sudo et al., 2004). Indeed, germ-free mice showed an enhanced HPA response to restraint stress, which was reversed by their re-conventionalization at early stages of development. According to the authors, both a cytokine-mediated humoral route and a neural mediated pathway could be involved in the microbiota modulation of the endocrine response in early life. Moreover, as observed by Diaz Heijtz et al. (2011) in mice, the GM can also affect the synaptogenesis during the perinatal period.

The recent use of germ-free and gnotobiotic mice has allowed the field to disentangle the complexity of the GM-host transgenomic metabolism, shedding light on the specific role of GM metabolites in host physiology (Nicholson et al., 2012). In particular, the endpoints of the GM polysaccharide fermentation, short-chain fatty acids (SCFAs) – most abundantly acetate, propionate, and butyrate – are key GM metabolites, with a multifactorial role in human health and homeostasis. These acids have been shown to play a pivotal role in host nutrition and energy homeostasis, controlling energy production, and storage as well as the appetite. Butyrate represents the main energetic substrate for the colonic epithelial cells (Russell et al., 2013) and both butyrate and propionate have been reported to activate intestinal gluconeogenesis (IGN) through two complementary mechanisms: butyrate triggers IGN gene expression in the gut via cAMP-dependent mechanisms, whereas propionate activates IGN by gene expression through gut-brain neural circuits and itself represents a substrate for IGN (De Vadder et al., 2014). According to the authors, this last propionate-dependent mechanism of IGN induction has been defined as strategic to provide the host with several metabolic benefits on body weight and glucose control. Besides the nutritional role, SCFAs have been reported to be involved in the maintenance of immune homeostasis (Arpaia et al., 2013). Through their production the GM controls the epithelium inflammation rate and drives the production and migration of specific immunological cells. Effectively, propionate governs the *de novo* peripheral regulatory T cell (Treg) generation and, together with acetate, drives their homing in the colon. Furthermore, propionate has been involved in the enhancement of hematopoiesis of dendritic cells with an impaired Th2 activation (Trompette et al., 2014). On the other hand, butyrate has the ability to regulate the production of pro-inflammatory cytokines (Segain et al., 2000), exerting a local immunomodulatory activity. Moreover, it is involved in the extrathymic Treg generation. In addition, the protective activity exerted by butyrate on the gut epithelium has been reported, as it stimulates the release of mucins (Petersson et al., 2011).

## GENETICS OF HOST AND MICROBIOTA

Host genetics and the GM are linked together by an intense cross-talk and this interaction is dynamic throughout the course of our life. Several studies have been conducted to determine the impact of host genetics on the GM composition providing conflicting results. To address how the host genotype and the environment influence the GM composition, a study on the fecal microbiota of monozygotic and dizygotic twin pairs concordant for leanness or obesity, and their mothers was conducted (Turnbaugh et al., 2009). The authors found that the GM of monozygotic twin pairs had a degree of similarity that was comparable to that of dizygotic twin pairs, highlighting that the environment impacts the GM composition more than the genetics does. It was also reported that family members harbor a similar GM composition and share a “core microbiome” made of several microbial genes. However, a study conducted on related and unrelated children reported that the highest level of similarity was found in genetically identical twins (Stewart et al., 2005).

Several studies reported that single host genes, i.e., MEditerranean FeVer, *APOA1, NOD2,* and *FUT2* affect the GM by altering its composition or by reducing the degree of bacterial diversity (Khachatryan et al., 2008; Petnicki-Ocwieja et al., 2009; Zhang et al., 2010; Frank et al., 2011; Wacklin et al., 2014).

Murine models proved to be very useful to clarify the effect of genetics on the GM. One of the first studies focused on the interaction host genetics-GM was based on observations on the GM reconstruction process occurring after a course of antibiotics (Vaahtovuo et al., 2003). It was observed that the colonization of the GM depends on the genotype of the host, and differences in communities between mouse strains were observed, supporting the idea that the gut community is not established by chance but is influenced by the host genetic background. Kovacs et al. (2011) studied particular recombinant inbred mouse strains to assess the relative role of the host genotype in the GM composition and they reported that the mouse genetic background is a strong determinant in shaping the intestinal microbiota. To address how environmental factors and host genetic factors combine to shape the GM, Benson et al. (2010) explored the associations between host quantitative trait loci (QTL) and the GM composition in mice. Eighteen host’s QTL showing a significant association with the relative abundance of specific microbial taxa were identified. Even if litter and cohort effects accounted for some of theGM variation, according to the authors host genetics had a greater impact on the GM variability.

All these studies provide information that supports the idea that the host genetics and the GM interact with each other deeply, and we can speculate that changes in the GM composition could boost the different genetic make up of every individual.

## MICROBIOTA ADAPTATION TO DAILY LIFE: MICROBIOME PLASTICITY IN RESPONSE TO DIFFERENT DIETS AND HOST PHYSIOLOGY

The human GM is a complex dynamic system with the potential for multistability. Indeed, Faith et al. (2013) found that on average 40% of the microbial strains harbored in an adult’s intestine was variable in a 5-year sampling period. In a mutualistic context the GM makes sudden jumps from different steady states under the pressure of environmental and endogenous factors, such as diet, age, host genetics, and physiological state (Candela et al., 2012).

The most rapid observable response of the gut microbial community is the reaction to daily dietary changes. Through a study where high-fat low-fiber and low-fat high-fiber diets were compared, changes in the microbiome composition were detected within 24 h of controlled feeding, confirming that the human gut ecosystem plasticity can respond efficiently and rapidly to external variables (Walker et al., 2011). However, as reported by Wu et al. (2011), short- and long-term dietary interventions differently impact the GM composition. Effectively, some bacterial groups are more likely to be influenced only by short-term dietary intervention, while others, namely those referred to as human enterotypes (Arumugam et al., 2011), are affected only by long-term intervention.

The correlation between nutrients and the GM composition was investigated in a caloric restriction study realized in 18 lean subjects over a 4-day period. The outcomes showed that nutritional compounds, like proteins and fibers, affect the phylogenetic and functional structure of the gut microbial community (Muegge et al., 2011). The connection between the GM phylogenetic profile and the ingestion of a specific nutrient, namely fermentable carbohydrate, was also observed in a recent study conducted in 14 overweight men in a 3-week period of intervention (Walker et al., 2011).

It has been recently demonstrated that animal-based and plant-based diets deeply impact on the GM (David et al., 2014). Both diets were administered for 5 days to 10 young US adults and the microbial community composition, metabolic products, and gene expression were analyzed. Interestingly, dietary changes to the plant- or animal-based diet resulted in marked microbiota changes only 1 day after the diet modification. In particular, the plant-based diet was associated with the presence of fibrolytic SCFA producers as *Roseburia*, *Eubacterium rectale*, and *Faecalibacterium prausnitzii*, while the animal-based diet resulted in the increase of potentially putrefactive microorganisms, such as *Bacteroides* and the bile tolerant *Bilophila wadsworthia* and *Alistipes*. It was observed that the animal-based diet had a greater impact on the GM structural and functional layout than the only plant-based diet. Lower levels of metabolic products resulting from the fermentation of carbohydrates and greater levels of the products resulting from the fermentation of amino acids were reported in individuals with the animal-based diet. Finally, the animal-based diet was associated with an increased expression of genes involved in the biosynthesis of vitamins and genes involved in the metabolism of products resulting from the consumption of meat (David et al., 2014). Interestingly, besides dietary substrates, the GM also relies on host-derived glycans secreted in the mucus as a nutrient source in the gut (Kashyap et al., 2013). Indeed, genetically dictated changes in host mucus glycan composition, such as the presence or absence of terminal fucose residues, have been shown to significantly impact the GM structure and function. This provides a global view where the diet and the host genotype interact to modulate the GM configuration.

The ability of the GM to re-program itself in response to different stimuli is necessary to adapt to the metabolic requirements of the host corresponding to different physiological states. For instance, pregnancy represents a period of deep physiological changes during which the GM composition adjusts according to the growth of the fetus and the lactation period. According to Koren et al. (2012), pregnancy is characterized by a greater inflammation tone, reduced insulin sensitivity, and body fat increase.

These traits are supported by a pregnancy-associated GM profile, whose main features are the expansion of Proteobacteria and Actinobacteria and a decrease in richness. Interestingly, such modifications in the GM composition persist for 1 month after birth and afterward the adult-like microbiota configuration is restored.

While the GM virtually varies in response to any changes in environmental and endogenous factors, the GM adaptation to extreme conditions – as an abnormal dietary sugar and fat intake or chronic inflammation – breaks the microbiota-host mutualistic homeostasis, lowering the ecosystem diversity, and overcoming the resilience of the microbiota-host symbiosis. The microbiota observed in obese people represents an appropriate example of unbalanced GM configuration driven by the Westernized diet and lifestyle (Ridaura et al., 2013). The functional annotation of the obese-type gut microbiome revealed a decreased functional diversity and an enrichment in genes involved in carbohydrate, lipid, and amino acid metabolism, showing an overall increased fermentative capacity with respect to the lean-type microbiome (Turnbaugh et al., 2009). Moreover, very recently, Schulz et al. (2014) demonstrated that a high-fat diet mediates shifts in the GM composition that promote intestinal carcinogenesis by compromising the Paneth-cell-mediated antimicrobial host defenses. On the other hand, inflammatory bowel disease (IBD) is a paradigmatic model to elucidate the self-sustained inflammatory loop that is established in the gut as a result of an inflammation-induced microbiota dysbiosis. In particular, inflammation forces GM to change toward a pro-inflammatory pathobionts-enriched profile, which consolidates the host inflammatory tone (Round and Mazmanian, 2009).

Therefore, the role of the GM as a plastic factor in response to environmental or endogenous stress is essential for the maintenance of the mutualistic relationship with the host. But under some specific circumstances the microbial community can be forced to shift to a disease-associated configuration with the breaking of the homeostasis balance.

## MICROBIOTA ADAPTATION TO DIFFERENT AGES

The human GM describes an evolutionary trajectory along the course of human life. The GM ecosystem changes its structural and functional layout from early infancy to old age, providing the host with ecosystem services finely calibrated for each stage of life (Yatsunenko et al., 2012). For instance, the peculiar GM composition during infancy exerts specific functions for the infant biology, supporting the immune system education, brain development, and host nutrition (Candela et al., 2013). At weaning, the GM gains diversity and develops new physiological functions, in order to fulfill the adult age-related requirements, such as the need to extract energy from the variable array of complex polysaccharides characterizing the adult diet.

The individual microbial layout begins to be formed immediately during delivery (Jost et al., 2012). We are born sterile and environmental microbes immediately colonize us (Palmer et al., 2007). The infant’ GIT is firstly colonized, just a few hours after birth, by facultative anaerobic bacteria, i.e., enterobacteria, staphylococci, and streptococci. Over time, the decreased amount of available oxygen allows strictly anaerobic bacteria to settle in the intestine, modifying the intestinal environment (Vael and Desager, 2009). In particular, Jost et al. (2012) analyzed the bacterial composition in feces from seven healthy vaginally delivered, breast-fed neonates at different times after birth. They observed that, during the first days of life, anaerobes, i.e., *Bifidobacterium* and *Bacteroides*, outnumbered facultative anaerobes in all seven neonates, pointing out that anaerobes may become dominant early in life and that the switch from facultative to strict anaerobes may occur at a very early stage. The infant-type microbiota is thus characterized by the dominance of *Bifidobacterium* and the presence of *Staphylococcus*, *Streptococcus,* and *Enterobacteriaceae* as other major components. With a relatively low degree of diversity, the infant-type GM is capable of tremendous fluctuations over time, with an individual-specific temporal pattern of variation in species composition (Palmer et al., 2007; Jost et al., 2012). The delivery mode is one of the factors that most influence early infants’ microbiota composition (Dominguez-Bello et al., 2010). Indeed, the authors observed that the vaginally delivered infants acquired bacterial communities resembling their own mother’s vaginal microbiota, while the cesarean section infants transiently harbored bacterial communities similar to those found on the mothers’ skin surface.

Largely dominated by *Bifidobacterium* and *Enterobacteriaceae* with an extraordinary rate of variation over time, the infant-type microbiota is functionally structured to educate the infant immune system through an intense, yet controlled, immunological dialog. Centanni et al. (2013) demonstrated that in infants the phylogenetic structure of the enterocyte-associated GM fraction was unaffected by the host inflammatory stimulus, probably because the GM of infants is specifically shaped to cope with the dynamic and intense cross-talk with the host immune system that is necessary for immune education. Recently, it has been shown that the diversity in the GM composition in infants is more important than the prevalence of specific bacterial taxa in the determination of the risk of immunological diseases later in life, i.e., allergic disease and asthma (Bisgaard et al., 2011; Abrahamsson et al., 2013). Furthermore, the infant GM also responds to precise developmental and nutritional needs crucial for the infant, such as the development and functionality of the central nervous system (Sudo et al., 2004; Collins et al., 2012), as well as the specific vitamin requests (Yatsunenko et al., 2012). Recently, fascinating hypotheses extending the GM-dependent immune and metabolic programming to the perinatal period have been advanced (Rautava et al., 2012). However, until confirmed by robust experimental findings, such hypotheses need to be taken with caution (Hanage, 2014).

The infant-type GM is subject to profound fluctuations until weaning when, with the introduction of solid food, it shifts toward the adult-type microbiota, with the progressive acquisition of taxonomic and functional complexity, such as a wide array of carbohydrate-active enzymes. This shift results in a profound change in the GM composition that goes from a bifidobacteria-enriched community to another one dominated by *Firmicutes* and *Bacteroidetes*, resembling more and more the microbiota of an adult, characterized by increased functionality and stability (Koenig et al., 2011). This adult-type microbiota is functionally structured to metabolize the whole complexity of the plant polysaccharides contained in the adult diet and provides mutual benefits to the host (Vanhoutte et al., 2004). Indeed, the microbiota takes advantage of a warm and nutrient-rich environment in which it can settle, while the host can benefit from an easy-fitting metabolic equipment that can provide essential factors and increase the host’s digestive capacity (Lozupone et al., 2012). A strong selection toward a readily changeable individual microbiome profile has been shown (Candela et al., 2012). This is the consequence of the inherent degree of plasticity of this bacterial ecosystem in adults, which allows the GM to change in response to environmental/endogenous factors, and the uniqueness of our physiology, lifestyle and history (Costello et al., 2012). These result in a peculiar temporal dynamics of the individual GM, always providing an adaptive response to ensure ecosystem services in the face of personalized physiology, immune system, environmental, or dietary exposure and lifestyle (Candela et al., 2013).

With aging and the onset of pathophysiological conditions (e.g., colon cancer, IBD, obesity, type 2 diabetes, and cardiovascular diseases) the GM-host mutualistic relationship progressively becomes compromised (Biagi et al., 2013). In elderly people diet and lifestyle undergo profound variations that include alterations of taste and smell, of gastrointestinal motility, and mastication, resulting in a nutritionally imbalanced diet (Biagi et al., 2010, 2013; Claesson et al., 2011; Drago et al., 2012). These age-related modifications, together with immunosenescence, affect the phylogenetic and functional structure of the gut ecosystem, leading to a microbial composition that favours the bloom of pathobionts (*Enterobacteriaceae*) to the detriment of immunomodulatory groups (*Clostridium* cluster IV and XIVa, *Bifidobacterium*). This age-associated configuration together with the “inflammaging” process could contribute to the creation of a self-sustained pro-inflammatory loop that is prejudicial for host health (Franceschi et al., 2000a,b; Grignolio et al., 2014). Interestingly, the GM of the elderly displays a restricted stability and extreme variability. Recently, a functional description of the aged GM was reported (Rampelli et al., 2013). By using Illumina shotgun sequencing, three centenarians’ fecal samples were analyzed and a shift from a saccharolytic to a putrefactive metabolism was reported. Indeed, an increase in the proteolytic potential, a reduction of genes involved in the metabolism of carbohydrates and a reduction of genes involved in SCFA production were observed. These modifications are in agreement with the age-related enrichment of genes belonging to pathobionts, and the authors hypothesized the existence of a pro-inflammatory loop in which pathobionts actively promote the worsening of health status with age. They also speculate that in centenarians some readjustments could occur to counteract the detrimental effects of pathobiont accumulation.

## MICROBIOTA ADAPTATION DURING DIETARY SHIFT IN HUMAN EVOLUTION

Bacteria are part of the evolutionary history of complex organisms and they occupy every ecological niche of our planet. The human GM is the biggest stable symbiont of our body (Costello et al., 2009) and it is characterized by a long adaptive history.

Modern humans, when have moved out of Africa, had to face different environmental challenges (such as food availability, climate changes, and pathogen loads). The main change in the host–microbiota symbiosis likely occurred almost 10,000 years ago, during the *Neolithic revolution*, also called “agricultural revolution” (De Filippo et al., 2010; Ottaviani et al., 2011). This revolution is based on the transition from hunting and gathering to agriculture and permanent settlements. In this period, the agriculture and animal husbandry have led to natural changes of human lifestyle and shaped modern human genomes. Given its high plasticity, the GM is able to change its composition and to adapt itself, according to diet/food availability, and the advent of agricultural societies could have favored microbial communities able to ferment complex substrates like polysaccharides (Hehemann et al., 2010). However, to date, little is known regarding how the GM has changed during human evolution. One of the most constraining aspects in this research field is the impossibility of having suitable fossil record. Indeed, the study of changes in the GM in human history is complicated by the difficulty in finding well-preserved samples of feces or intestinal samples of different periods (Walter and Ley, 2011). Nevertheless, researchers are developing methods to overcome this limitation. In a very recent paper, Sistiaga et al. (2014) applied gas-chromatography-mass spectrometry to Neanderthal’s fecal matter to evaluate sterol and stanol level. The authors provide the first evidence that, even if Neanderthals predominantly consumed meat, they also had a remarkable plant intake, and they suggest the presence of a specific GM involved in cholesterol metabolism throughout human evolution. On the other hand, a glimpse of the ancestral human GM configuration could be provided by the GM of close primate relatives (Ley et al., 2008b; Moeller et al., 2012). Interestingly, the GM of modern humans clusters with that of other omnivorous primates, regardless of their affiliation to *Pan* (Ley et al., 2008b). This supports the key role of dietary habits in shaping the composition of the GIT microbial ecosystem.

**FIGURE 1:**
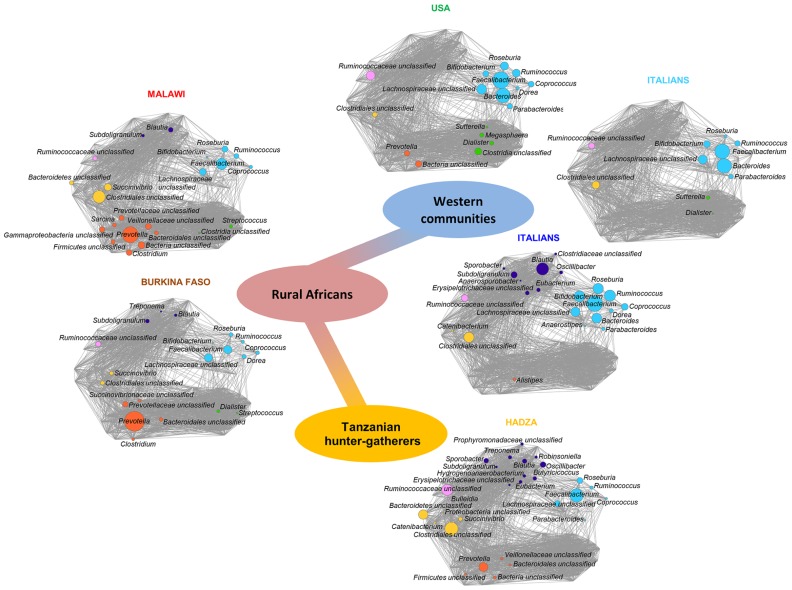
** Timescale of the intestinal microbiota evolution: from foraging to Western lifestyle, crossing the Neolithic revolution.** The trajectory of the gut microbiome structure of modern populations with different lifestyles mimics the evolution of the relationship between microbes and the human host. Each network plot is a Wiggum plot, published in Schnorr et al. (2014), which indicates patterns of variation of six identified co-abundance groups (CAGs) in the Hadza (orange; Schnorr et al., 2014), Malawi (red; Yatsunenko et al., 2012), Burkina Faso (brown; De Filippo et al., 2010), Italians (blue; Schnorr et al., 2014), US people (green; Yatsunenko et al., 2012), and Italian children (cyan; De Filippo et al., 2010). CAGs are named by the name of the most abundant genera and are color coded as follows: *Faecalibacterium* (cyan), *Dialister* (green), *Prevotella* (orange), *Clostridiales*_unclassified (yellow), *Ruminococcaceae*_unclassified (pink), and *Blautia* (violet). Each node represents a bacterial genus and its dimension is proportional to the mean relative abundance within the population. Connections between nodes represent positive and significant Kendall correlations between genera (false discovery rate <0.05). The central path indicates transition from hunter-gatherer (orange) to Western microbiome (blue), crossing the rural African configuration (red).

The dynamics of the GM-host co-evolution and environmental adaptation can be addressed by investigating the GM variability in modern human populations of different culture (Candela et al., 2012). Indeed, the study of the GM from large healthy human populations of different age and socio-economic, geographic, and cultural settings allows researchers to point out the contribution of these environment components to the GM variation. In this context, a very recent paper explored the GM of the Hadza of Tanzania, a modern population of hunter-gatherers that still live as Paleolithic humans (Schnorr et al., 2014). This study elucidated the mechanisms of humans/GM co-evolution and showed a first map of the microbiota composition of the Hadza that reflects the functional adaptation to a foraging lifestyle. For instance, the high bacterial diversity and the enrichment in fibrolytic microorganisms (e.g., xylan-degrading *Prevotella* and *Treponema*) proper of the Hadza GM, represent ecosystem adaptations to provide SCFAs from their heavy plant-based diet. Furthermore, the Hadza show a sex-related divergence in the GM composition reflecting the sexual division of labor and sex differences in diet composition. In particular, the higher relative abundance of *Treponema* found in Hadza women could provide specific functions to deal with their higher intake of tubers and plant foods. In fact, women selectively forage for tubers and plant foods and spend a lot of time in camp, while men are highly mobile foragers and range far from the central camp site to obtain meat and honey. Even if foods are brought back to the camp and shared, men and women tend to consume more of their targeted foods. Finally, the absence of *Bifidobacterium* and a corresponding enrichment of potential opportunists as Proteobacteria and Spirochaetes in the Hadza GM probably correspond to a different tolerogenic layout of their immune system, redefining the notion of what we consider a healthy and an unhealthy GM structure. Indeed, the Hadza have relatively low rates of infectious diseases, metabolic diseases and nutritional deficiencies in comparison with other groups settled in Northern Tanzania (Bennett et al., 1973; Work et al., 1973; Blurton Jones et al., 1992). Moreover, De Filippo et al. (2010) compared the GM of children living in rural Africa and that of European children, and many differences emerged. Children from Boulpon Rural Village in Burkina Faso have a traditional rural African diet that is rich in starch, fibers, and plant polysaccharides and low in fat and animal protein, while European children follow a Western diet. The authors argued that the consumption of sugar, animal fat, and calorie-dense foods in industrialized countries is rapidly limiting the adaptive potential of the microbiota, by reducing microbiota richness and its functionality. Interestingly, the authors reported that only the GM of African children contains *Prevotella*, *Xylanibacter,* and *Treponema* that are involved in cellulose and xylan hydrolysis. It was speculated that the high fiber intake characterizing the African diet could change the GM composition to maximize the metabolic energy extraction from ingested plant polysaccharides (De Filippo et al., 2010). Finally, the same approach was applied to fecal samples from 531 children and adults from the Amazonas of Venezuela, rural Malawi and US metropolitan areas, including parents, siblings, and twins (Yatsunenko et al., 2012). The phylogenetic composition of the GM of these three populations is different, especially for US residents vs. non-US residents (Malawians and Amerindians). Furthermore, the authors confirmed the importance of *Prevotella* as a discriminatory taxon that distinguishes non-US from US individuals. A meta-analysis of the GM composition in Western populations (i.e., USA and Italian citizens), rural Malawi and Burkina Faso populations, and Hadza hunter-gatherers has been carried out (Schnorr et al., 2014). Data allowed to reconstruct the putative trajectory of GM adaptive evolution that accompanied human beings along the transition from the Paleolithic hunter-gatherer to the Neolithic rural communities until modern Western societies. The diagram reported in **Figure 1** shows the emergence of specific co-abundance groups (CAGs) – groups of microorganisms which correlate and cluster together – along with the most important transition phases in our recent evolutionary history, such as the higher abundance of *Ruminococcaceae* unclassified CAG distinguishing for the Hadza hunter-gatherers, the emergence of *Clostridiales* unclassified and *Prevotella* CAGs in rural Malawi and Burkina Faso populations, and the dominance of the *Faecalibacterium* CAG in Western populations (Schnorr et al., 2014).

A striking and fascinating example for gene acquisition by a gut microbe as an adaptation to the local diet is described by Hehemann et al. (2010) in a study on Japanese population. By comparing the GM from Japanese and North American populations, it was reported that the GM of Japanese subjects is enriched for genes (probably acquired by contact with marine microbes) that encode enzymes capable of degrading porphyran that is contained in seaweeds. In the Japanese culture, the greatest source of porphyran is due to nori (edible seaweed), which is commonly used in the preparation of sushi and it is the most produced and consumed seaweed for centuries in Japan.

## CONCLUSION

Human beings faced tremendous changes in lifestyle and dietary habits along the course of their recent evolutionary history. They passed from Paleolithic hunter-gathering societies to Neolithic agricultural farmers until modern Westernized societies in 10,000 years, adapting to dramatic changes in diet and lifestyle in a relatively short evolutionary frame.

Human beings have been recently revised as superorganisms as a result of a close mutualistic relationship with their GM. Recent longitudinal studies highlighted an adaptive role for the GM in human biology, allowing to optimize the superorganism metabolic performances in response to diet, lifestyle, and physiological changes such as aging. This raises the question of whether this adaptive GM potential had a role in our recent evolutionary history, allowing adaptation to the profound lifestyle changes and describing a real GM-host evolutionary trajectory.

The first and very recent description of the Hadza GM structure provided important light in this direction. The peculiar structural and functional configuration of the Hadza gut microbial ecosystem suggests that adaptive functional changes of the GM accompanied the evolutionary trajectory of human beings, allowing the optimization of the superorganism performances in response to the profound changes that characterized our recent evolutionary history.

However, several circumstances characteristic of the Western world are challenging the resilience of the GM-host mutualistic interaction. The high-fat high-sugar energy-dense diet, sanitization, and antibiotic usage – a landmark of Western societies – are forcing GM adaptive changes to deviate from a mutualistic configuration. This raises the need to better comprehend the dynamics involved in this process, controlling any variables to preserve the extraordinary mutualistic relationship we evolved with our microbial counterpart.

## Conflict of Interest Statement

The authors declare that the research was conducted in the absence of any commercial or financial relationships that could be construed as a potential conflict of interest.

## References

[B1] Abrahamsson T. R. Jakobsson H. E. Andersson A. F. Björkstén B. Engstrand L. Jenmalm M. C. (2013). Low gut microbiota diversity in early infancy precedes asthma at school age. *Clin. Exp. Allergy* 44 842–850 10.1111/cea.12253 24330256

[B2] Arpaia N. Campbell C. Fan X. Dikiy S. van der Veeken J. deRoos P. (2013). Metabolites produced by commensal bacteria promote peripheral regulatory T-cell generation. *Nature* 504 451–455 10.1038/nature12726 24226773 PMC3869884

[B3] Arumugam M. Raes J. Pelletier E. Le Paslier D. Yamada T. Mende D. R. (2011). Enterotypes of the human gut microbiome. *Nature* 473 174–180 10.1038/nature09944 21508958 PMC3728647

[B4] Bäckhed F. Ding H. Wang T. Hooper L. V. Koh G. Y. Nagy A. (2004). The gut microbiota as an environmental factor that regulates fat storage. *Proc. Natl. Acad. Sci. U.S.A.* 101 15718–15723 10.1073/pnas.0407076101 15505215 PMC524219

[B5] Bennett F. J. Barnicot N. A. Woodburn J. C. Pereira M. S. Henderson B. E. (1973). Studies on viral, bacterial, rickettsial and treponemal diseases in the hadza of Tanzania and a note on injuries. *Hum. Biol.* 45 243–272. 4740327

[B6] Benson A. K. Kelly S. A. Legge R. Ma F. Low S. J. Kim J. (2010). Individuality in gut microbiota composition is a complex polygenic trait shaped by multiple environmental and host genetic factors. *Proc. Natl. Acad. Sci. U.S.A.* 107 18933–18938 10.1073/pnas.1007028107 20937875 PMC2973891

[B7] Biagi E. Candela M. Turroni S. Garagnani P. Franceschi C. Brigidi P. (2013). Ageing and gut microbes: perspectives for health maintenance and longevity. *Pharmacol. Res.* 69 11–20 10.1016/j.phrs.2012.10.005 23079287

[B8] Biagi E. Nylund L. Candela M. Ostan R. Bucci L. Pini E. (2010). Through ageing, and beyond: gut microbiota and inflammatory status in seniors and centenarians. *PLoS ONE* 5:e10667 10.1371/journal.pone.0010667 PMC2871786 20498852

[B9] Bisgaard H. Li N. Bonnelykke K. Chawes B. L. Skov T. Paludan-Müller G. (2011). Reduced diversity of the intestinal microbiota during infancy is associated with increased risk of allergic disease at school age. *J. Allergy Clin. Immunol.* 128 646–652 e1–5 10.1016/j.jaci.2011.04.060 21782228

[B10] Blurton Jones N. G. Smith L. C. O’Connell J. F. Hawkes K. Kamuzora C. L. (1992). Demography of the hadza, an increasing and high density population of savanna foragers. *Am. J. Phys. Anthropol.* 89 159–181 10.1002/ajpa.1330890204 1443092

[B11] Candela M. Biagi E. Maccaferri S. Turroni S. Brigidi P. (2012). Intestinal microbiota is a plastic factor responding to environmental changes. *Trends Microbiol.* 20 385–391 10.1016/j.tim.2012.05.003 22672911

[B12] Candela M. Biagi E. Turroni S. Maccaferri S. Figini P. Brigidi P. (2013). Dynamic efficiency of the human intestinal microbiota. *Crit. Rev. Microbiol.* 1–7 10.3109/1040841X.2013.813900 25168339

[B13] Centanni M. Turroni S. Consolandi C. Rampelli S. Peano C. Severgnini M. (2013). The enterocyte-associated intestinal microbiota of breast-fed infants and adults responds differently to a TNF-a-mediated pro-inflammatory stimulus. *PLoS ONE* 8:e81762 10.1371/journal.pone.0081762 PMC3841132 24303069

[B14] Claesson M. J. Cusack S. O’Sullivan O. Greene-Diniz R. de Weerd H. Flannery E. (2011). Composition, variability, and temporal stability of the intestinal microbiota of the elderly. *Proc. Natl. Acad. Sci. U.S.A.* 108(Suppl. 1) 4586–4591 10.1073/pnas.1000097107 20571116 PMC3063589

[B15] Clemente J. C. Ursell L. K. Parfrey L. W. Knight R. (2012). The impact of the gut microbiota on human health: an integrative view. *Cell* 148 1258–1270 10.1016/j.cell.2012.01.035 22424233 PMC5050011

[B16] Collins S. M. Surette M. Bercik P. (2012). The interplay between the intestinal microbiota and the brain. *Nat. Rev. Microbiol.* 10 735–742 10.1038/nrmicro2876 23000955

[B17] Costello E. K. Lauber C. L. Hamady M. Fierer N. Gordon J. I. Knight R. (2009). Bacterial community variation in human body habitats across space and time. *Science* 326 1694–1697 10.1126/science.1177486 19892944 PMC3602444

[B18] Costello E. K. Stagaman K. Dethlefsen L. Bohannan B. J. Relman D. A. (2012). The application of ecological theory toward an understanding of the human microbiome. *Science* 336 1255–1262 10.1126/science.1224203 22674335 PMC4208626

[B19] Cryan J. F. Dinan T. G. (2012). Mind-altering microorganisms: the impact of the gut microbiota on brain and behaviour. *Nat. Rev. Neurosci.* 13 701–712 10.1038/nrn3346 22968153

[B20] David L. A. Maurice C. F. Carmody R. N. Gootenberg D. B. Button J. E. Wolfe B. E. (2014). Diet rapidly and reproducibly alters the human gut microbiome. *Nature* 505 559–563 10.1038/nature12820 24336217 PMC3957428

[B21] De Filippo C. Cavalieri D. Di Paola M. Ramazzotti M. Poullet J. B. Massart S. (2010). Impact of diet in shaping gut microbiota revealed by a comparative study in children from europe and rural Africa. *Proc. Natl. Acad. Sci. U.S.A.* 107 14691–14696 10.1073/pnas.1005963107 20679230 PMC2930426

[B22] De Vadder F. Kovatcheva-Datchary P. Goncalves D. Vinera J. Zitoun C. Duchampt A. (2014). Microbiota-generated metabolites promote metabolic benefits via gut-brain neural circuits. *Cell* 156 84–96 10.1016/j.cell.2013.12.016 24412651

[B23] Diaz Heijtz R. Wang S. Anuar F. Qian Y. Björkholm B. Samuelsson A. (2011). Normal gut microbiota modulates brain development and behavior. *Proc. Natl. Acad. Sci. U.S.A.* 108 3047–3052 10.1073/pnas.1010529108 21282636 PMC3041077

[B24] Dominguez-Bello M. G. Costello E. K. Contreras M. Magris M. Hidalgo G. Fierer N. (2010). Delivery mode shapes the acquisition and structure of the initial microbiota across multiple body habitats in newborns. *Proc. Natl. Acad. Sci. U.S.A.* 107 11971–11975 10.1073/pnas.1002601107 20566857 PMC2900693

[B25] Drago L. Toscano M. Rodighiero V. De Vecchi E. Mogna G. (2012). Cultivable and pyrosequenced fecal microflora in centenarians and young subjects. *J. Clin. Gastroenterol.* 46(Suppl. 1) S81–S84 10.1097/MCG.0b013e3182693982 22955365

[B26] Faith J. J. Guruge J. L. Charbonneau M. Subramanian S. Seedorf H. Goodman A. L. (2013). The long-term stability of the human gut microbiota. *Science* 341:1237439 10.1126/science.1237439 PMC3791589 23828941

[B27] Foster J. A. McVey Neufeld K.-A. (2013). Gut-brain axis: how the microbiome influences anxiety and depression. *Trends Neurosci.* 36 305–312 10.1016/j.tins.2013.01.005 23384445

[B28] Franceschi C. Bonafè M. Valensin S. Olivieri F. De Luca M. Ottaviani E. (2000a). Inflamm-aging. an evolutionary perspective on immunosenescence. *Ann. N. Y. Acad. Sci.* 908 244–54 10.1111/j.1749-6632.2000.tb06651.x 10911963

[B29] Franceschi C. Valensin S. Bonafè M. Paolisso G. Yashin A. I. Monti D. (2000b). The network and the remodeling theories of aging: historical background and new perspectives. *Exp. Gerontol.* 35 879–896 10.1016/S0531-5565(00)00172-8 11053678

[B30] Frank D. N. Robertson C. E. Hamm C. M. Kpadeh Z. Zhang T. Chen H. (2011). Disease phenotype and genotype are associated with shifts in intestinal-associated microbiota in inflammatory bowel diseases. *Inflamm. Bowel Dis.* 17 179–184 10.1002/ibd.21339 20839241 PMC3834564

[B31] Gill S. R. Pop M. Deboy R. T. Eckburg P. B. Turnbaugh P. J. Samuel B. S. (2006). Metagenomic analysis of the human distal gut microbiome. *Science* 312 1355–1359 10.1126/science.1124234 16741115 PMC3027896

[B32] Grignolio A. Mishto M. Faria A. M. Garagnani P. Franceschi C. Tieri P. (2014). Towards a liquid self: how time, geography, and life experiences reshape the biological identity. *Front. Immunol.* 5:153 10.3389/fimmu.2014.00153 PMC3988364 24782860

[B33] Hanage W. P. (2014). Microbiology: microbiome science needs a healthy dose of scepticism. *Nature* 512 247–248 10.1038/512247a 25143098

[B34] Hehemann J.-H. Correc G. Barbeyron T. Helbert W. Czjzek M. Michel G. (2010). Transfer of carbohydrate-active enzymes from marine bacteria to japanese gut microbiota. *Nature* 464 908–912 10.1038/nature08937 20376150

[B35] Jost T. Lacroix C. Braegger C. P. Chassard C. (2012). New insights in gut microbiota establishment in healthy breast fed neonates. *PLoS ONE* 7:e44595 10.1371/journal.pone.0044595 PMC3431319 22957008

[B36] Kamada N. Seo S.-U. Chen G. Y. Núñez G. (2013). Role of the gut microbiota in immunity and inflammatory disease. *Nat. Rev. Immunol.* 13 321–335 10.1038/nri3430 23618829

[B37] Kashyap P. C. Marcobal A. Ursell L. K. Smits S. A. Sonnenburg E. D. Costello E. K. (2013). Genetically dictated change in host mucus carbohydrate landscape exerts a diet-dependent effect on the gut microbiota. *Proc. Natl. Acad. Sci. U.S.A.* 110 17059–17064 10.1073/pnas.1306070110 24062455 PMC3800993

[B38] Khachatryan Z. A. Ktsoyan Z. A. Manukyan G. P. Kelly D. Ghazaryan K. A. Aminov R. I. (2008). Predominant role of host genetics in controlling the composition of gut microbiota. *PLoS ONE* 3:e3064 10.1371/journal.pone.0003064 PMC2516932 18725973

[B39] Koenig J. E. Spor A. Scalfone N. Fricker A. D. Stombaugh J. Knight R. (2011). Succession of microbial consortia in the developing infant gut microbiome. *Proc. Natl. Acad. Sci.* 108(Suppl. 1) 4578–4585 10.1073/pnas.1000081107 20668239 PMC3063592

[B40] Koren O. Goodrich J. K. Cullender T. C. Spor A. Laitinen K. Bäckhed H. K. (2012). Host remodeling of the gut microbiome and metabolic changes during pregnancy. *Cell* 150 470–480 10.1016/j.cell.2012.07.008 22863002 PMC3505857

[B41] Koropatkin N. M. Cameron E. A. Martens E. C. (2012). How glycan metabolism shapes the human gut microbiota. *Nat. Rev. Microbiol.* 10 323–335 10.1038/nrmicro2746 22491358 PMC4005082

[B42] Kovacs A. Ben-Jacob N. Tayem H. Halperin E. Iraqi F. A. Gophna U. (2011). Genotype is a stronger determinant than sex of the mouse gut microbiota. *Microb. Ecol.* 61 423–428 10.1007/s00248-010-9787-2 21181142

[B43] Ley R. E. Lozupone C. A. Hamady M. Knight R. Gordon J. I. (2008a). Worlds within worlds: evolution of the vertebrate gut microbiota. *Nat. Rev. Microbiol.* 6 776–788 10.1038/nrmicro1978 18794915 PMC2664199

[B44] Ley R. E. Hamady M. Lozupone C. Turnbaugh P. J. Ramey R. R. Bircher J. S. (2008b). Evolution of mammals and their gut microbes. *Science* 320 1647–1651 10.1126/science.1155725 18497261 PMC2649005

[B45] Lozupone C. A. Stombaugh J. I. Gordon J. I. Jansson J. K. Knight R. (2012). Diversity, stability and resilience of the human gut microbiota. *Nature* 489 220–230 10.1038/nature11550 22972295 PMC3577372

[B46] Maynard C. L. Elson C. O. Hatton R. D. Weaver C. T. (2012). Reciprocal interactions of the intestinal microbiota and immune system. *Nature* 489 231–241 10.1038/nature11551 22972296 PMC4492337

[B47] Moeller A. H. Degnan P. H. Pusey A. E. Wilson M. L. Hahn B. H. Ochman H. (2012). Chimpanzees and humans harbour compositionally similar gut enterotypes. *Nat. Commun.* 3:1179 10.1038/ncomms2159 PMC3520023 23149725

[B48] Muegge B. D. Kuczynski J. Knights D. Clemente J. C. González A. Fontana L. (2011). Diet drives convergence in gut microbiome functions across mammalian phylogeny and within humans. *Science* 332 970–974 10.1126/science.1198719 21596990 PMC3303602

[B49] Nicholson J. K. Holmes E. Kinross J. Burcelin R. Gibson G. Jia W. (2012). Host-gut microbiota metabolic interactions. *Science* 336 1262–1267 10.1126/science.1223813 22674330

[B50] O’Hara A. M. Shanahan F. (2006). The gut flora as a forgotten organ. *EMBO Rep.* 7 688–693 10.1038/sj.embor.7400731 16819463 PMC1500832

[B51] Ottaviani E. Ventura N. Mandrioli M. Candela M. Franchini A. Franceschi C. (2011). Gut microbiota as a candidate for lifespan extension: an ecological/evolutionary perspective targeted on living organisms as metaorganisms. *Biogerontology* 12 599–609 10.1007/s10522-011-9352-5 21814818

[B52] Palmer C. Bik E. M. DiGiulio D. B. Relman D. A. Brown P. O. (2007). Development of the human infant intestinal microbiota. *PLoS Biol.* 5:e177 10.1371/journal.pbio.0050177 PMC1896187 17594176

[B53] Petersson J. Schreiber O. Hansson G. C. Gendler S. J. Velcich A. Lundberg J. O. (2011). Importance and regulation of the colonic mucus barrier in a mouse model of colitis. *Am. J. Physiol. Gastrointest. Liver Physiol.* 300 G327–G333 10.1152/ajpgi.00422.2010 21109593 PMC3302190

[B54] Petnicki-Ocwieja T. Hrncir T. Liu Y.-J. Biswas A. Hudcovic T. Tlaskalova-Hogenova H. (2009). Nod2 is required for the regulation of commensal microbiota in the intestine. *Proc. Natl. Acad. Sci. U.S.A.* 106 15813–15818 10.1073/pnas.0907722106 19805227 PMC2747201

[B55] Qin J. Li R. Raes J. Arumugam M. Burgdorf K. S. Manichanh C. (2010). A human gut microbial gene catalogue established by metagenomic sequencing. *Nature* 464 59–65 10.1038/nature08821 20203603 PMC3779803

[B56] Rampelli S. Candela M. Turroni S. Biagi E. Collino S. Franceschi C. (2013). Functional metagenomic profiling of intestinal microbiome in extreme ageing. *Aging (Albany, NY)* 5 902–912. 24334635 10.18632/aging.100623 PMC3883706

[B57] Rautava S. Luoto R. Salminen S. Isolauri E. (2012). Microbial contact during pregnancy, intestinal colonization and human disease. *Nat. Rev. Gastroenterol. Hepatol.* 9 565–576 10.1038/nrgastro.2012.144 22890113

[B58] Ridaura V. K. Faith J. J. Rey F. E. Cheng J. Duncan A. E. Kau A. L. (2013). Gut microbiota from twins discordant for obesity modulate metabolism in mice. *Science* 341:1241214 10.1126/science.1241214 PMC3829625 24009397

[B59] Round J. L. Mazmanian S. K. (2009). The gut microbiota shapes intestinal immune responses during health and disease. *Nat. Rev. Immunol.* 9 313–323 10.1038/nri2515 19343057 PMC4095778

[B60] Russell W. R. Hoyles L. Flint H. J. Dumas M.-E. (2013). Colonic bacterial metabolites and human health. *Curr. Opin. Microbiol.* 16 246–254 10.1016/j.mib.2013.07.002 23880135

[B61] Schnorr S. L. Candela M. Rampelli S. Centanni M. Consolandi C. Basaglia G. (2014). Gut microbiome of the hadza hunter-gatherers. *Nat. Commun.* 5:3654 10.1038/ncomms4654 PMC3996546 24736369

[B62] Schulz M. D. Atay C. Heringer J. Romrig F. K. Schwitalla S. Aydin B. (2014). High-fat-diet-mediated dysbiosis promotes intestinal carcinogenesis independently of obesity. *Nature* 514 508–512 10.1038/nature13398 25174708 PMC4233209

[B63] Segain J. P. Raingeard de la Blétière D. Bourreille A. Leray V. Gervois N. Rosales C. (2000). Butyrate inhibits inflammatory responses through NFkappaB inhibition: implications for Crohn’s Disease. *Gut* 47 397–403 10.1136/gut.47.3.397 10940278 PMC1728045

[B64] Sistiaga A. Mallol C. Galván B. Summons R. E. (2014). The eanderthal meal: a new perspective using faecal biomarkers. *PLoS ONE* 9:e101045 10.1371/journal.pone.0101045 PMC4071062 24963925

[B65] Stewart J. A. Chadwick V. S. Murray A. (2005). Investigations into the influence of host genetics on the predominant eubacteria in the faecal microflora of children. *J. Med. Microbiol.* 54 1239–1242 10.1099/jmm.0.46189-0 16278440

[B66] Sudo N. Chida Y. Aiba Y. Sonoda J. Oyama N. Yu X.-N. (2004). Postnatal microbial colonization programs the hypothalamic-pituitary-adrenal system for stress response in mice. *J. Physiol.* 558 263–275 10.1113/jphysiol.2004.063388 15133062 PMC1664925

[B67] Trompette A. Gollwitzer E. S. Yadava K. Sichelstiel A. K. Sprenger N. Ngom-Bru C. (2014). Gut microbiota metabolism of dietary fiber influences allergic airway disease and hematopoiesis. *Nat. Med.* 20 159–166 10.1038/nm.3444 24390308

[B68] Turnbaugh P. J. Hamady M. Yatsunenko T. Cantarel B. L. Duncan A. Ley R. E. (2009). A core gut microbiome in obese and lean twins. *Nature* 457 480–484 10.1038/nature07540 19043404 PMC2677729

[B69] Turnbaugh P. J. Ley R. E. Hamady M. Fraser-Liggett C. M. Knight R. Gordon J. I. (2007). The human microbiome project. *Nature* 449 804–810 10.1038/nature06244 17943116 PMC3709439

[B70] Vaahtovuo J. Toivanen P. Eerola E. (2003). Bacterial composition of murine fecal microflora is indigenous and genetically guided. *FEMS Microbiol. Ecol.* 44 131–136 10.1016/S0168-6496(02)00460-9 19719658

[B71] Vael C. Desager K. (2009). The importance of the development of the intestinal microbiota in infancy. [Miscellaneous Article]. *Curr. Opin. Pediatrics* 21 794–800 10.1097/MOP.0b013e328332351b 19770768

[B72] Vanhoutte T. Huys G. Brandt E. Swings J. (2004). Temporal stability analysis of the microbiota in human feces by denaturing gradient gel electrophoresis using universal and group-specific 16S rRNA gene primers. *FEMS Microbiol. Ecol.* 48 437–446 10.1016/j.femsec.2004.03.001 19712312

[B73] Wacklin P. Tuimala J. Nikkilä J. Tims S. Mäkivuokko H. Alakulppi N. (2014). Faecal microbiota composition in adults is associated with the FUT2 gene determining the secretor status. *PLoS ONE* 9:e94863 10.1371/journal.pone.0094863 PMC3986271 24733310

[B74] Walker A. W. Ince J. Duncan S. H. Webster L. M. Holtrop G. Ze X. (2011). Dominant and diet-responsive groups of bacteria within the human colonic microbiota. *ISME J.* 5 220–230 10.1038/ismej.2010.118 20686513 PMC3105703

[B75] Walter J. Ley R. (2011). The human gut microbiome: ecology and recent evolutionary changes. *Ann. Rev. Microbiol.* 65 411–429 10.1146/annurev-micro-090110-102830 21682646

[B76] Work T. H. Ifekwunigwe A. Jelliffe D. B. Jelliffe P. Neumann C. G. (1973). Tropical problems in nutrition. *Ann. Intern. Med.* 79 701–711 10.7326/0003-4819-79-5-701 4201635

[B77] Wu G. D. Chen J. Hoffmann C. Bittinger K. Chen Y.-Y. Keilbaugh S. A. (2011). Linking long-term dietary patterns with gut microbial enterotypes. *Science* 334 105–108 10.1126/science.1208344 21885731 PMC3368382

[B78] Yatsunenko T. Rey F. E. Manary M. J. Trehan I. Dominguez-Bello M. G. Contreras M. (2012). Human gut microbiome viewed across age and geography. *Nature* 486 222–227 10.1038/nature11053 22699611 PMC3376388

[B79] Zhang C. Zhang M. Wang S. Han R. Cao Y. Hua W. (2010). Interactions between gut microbiota, host genetics and diet relevant to development of metabolic syndromes in mice. *ISME J.* 4. 232–241 10.1038/ismej.2009.112 19865183

